# The amount of femorotibial alignment correction through total knee arthroplasty may affect postoperative hindfoot alignment

**DOI:** 10.1002/jeo2.12066

**Published:** 2024-06-19

**Authors:** Tsutomu Nakayama, Sachiyuki Tsukada, Yuya Kagami, Shingo Numajiri, Kenji Kurosaka, Masahiro Nishino, Naoyuki Hirasawa

**Affiliations:** ^1^ Department of Rehabilitation Hokusuikai Kinen Hospital Mito Ibaraki Japan; ^2^ Department of Orthopaedic Surgery Hokusuikai Kinen Hospital Mito Ibaraki Japan; ^3^ Department of Orthopaedic Surgery Tokyo Orthopedic Knee Hip Clinic Tokyo Japan

**Keywords:** alignment, arthroplasty, hindfoot, knee

## Abstract

**Purpose:**

This study was performed to investigate the relationship between the amount of femorotibial alignment correction and the amount of improvement of hindfoot alignment in total knee arthroplasty (TKA).

**Methods:**

A total of 159 knees undergoing TKA in 120 patients were assessed preoperatively and at 2 weeks, 1 month, 3 months and 6 months postoperatively. Standing hindfoot alignment was evaluated using the leg‐heel angle (LHA). The amount of change in hindfoot alignment was compared between patients with severe varus knee (Group 1) and those with moderate varus, neutral or valgus knee (Group 2).

**Results:**

The mean values of pre‐ and postoperative hip‐knee‐ankle (HKA) angle were −14 ± 4° and −1 ± 3° in Group 1 and −7 ± 5° and −1 ± 3° in Group 2, respectively. The differences between pre‐ and postoperative LHA were significantly larger in Group 1 than in Group 2 at 2 weeks, 1 month and 3 months postoperatively (*p* = 0.006, 0.001 and 0.03, respectively). At 6 months postoperatively, no differences were observed between the two groups (*p* = 0.31).

**Conclusion:**

The amount of change in hindfoot alignment was larger in Group 1 than in Group 2 at 2 weeks, 1 month and 3 months after TKA, but there was no significant difference between the two groups at 6 months after TKA.

**Level of Evidence:**

Prognostic Level II.

**Abbreviations**
CI: confidence interval
HKA angle: hip‐knee‐ankle angle
KOOS: knee injury and osteoarthritis outcome score
LHA: leg‐heel angle
TKA: total knee arthroplasty

## INTRODUCTION

Change of tibiofemoral alignment at the knee joint level after total knee arthroplasty (TKA) is associated with hindfoot alignment [11]. Many studies have shown that neutralizing varus femorotibial alignment with TKA can also neutralize valgus hindfoot alignment [1, 2, 3, 4, 5, 6, 10, 11, 12, 13].

In patients of severe preoperative varus femorotibial alignment, a greater amount of alignment correction is required at the level of the knee joint in TKA. There have been conflicting reports about the relationship between the amount of alignment correction and the improvement of hindfoot alignment [1, 2, 3, 4, 6, 10]. Some studies showed that the change in hindfoot alignment was correlated with the change in femorotibial alignment [2, 3, 6, 10], whereas others showed no such relationship [1, 4]. In addition, few investigators conducted direct interviews with patients regarding complaints about the hindfoot after TKA [7]. Clarifying how femorotibial alignment correction through TKA is related to postoperative hindfoot alignment would allow both surgeons and patients to anticipate potential complaints regarding the hindfoot following surgery.

The purpose of this study is to investigate the relationship between the amount of femorotibial alignment correction and the amount of improvement of hindfoot alignment in TKA. The hypotheses of this study were: (1) there is a correlation between the amount of tibiofemoral alignment correction and the amount of hindfoot alignment improvement; and (2) there is a relationship between the amount of tibiofemoral alignment correction and the occurrence of hindfoot complaints.

## PATIENTS AND METHODS

This study was performed following institutional review board approval. Patients who underwent TKA from January 2021 and May 2021 in a single institution were prospectively assessed. The exclusion criteria were revision knee arthroplasty, postoperative complications, rigid ankle deformities, ankle trauma history and correction of severe valgus knees (hip‐knee‐ankle [HKA] angle exceeding 10°). All patients provided written informed consent. In principle, the positioning of both tibial and femoral prostheses was planned perpendicular to the mechanical axis in all TKAs; in proximal tibia osteotomy, the cutting surface was aimed to be perpendicular to the tibial axis in both coronal and sagittal planes. The distal femoral osteotomy was made in valgus angulation, which was equal to the angle between the anatomical and functional axes of the femur [14]. All patients were evaluated preoperatively and at 2 weeks, 1 month, 3 months and 6 months postoperatively. Pre‐ and postoperative standing long‐leg radiographs were taken in all patients to evaluate the coronal alignment of the patient's leg. The radiographs were taken with the patella facing forward.

### Outcomes

**Figure 1:**
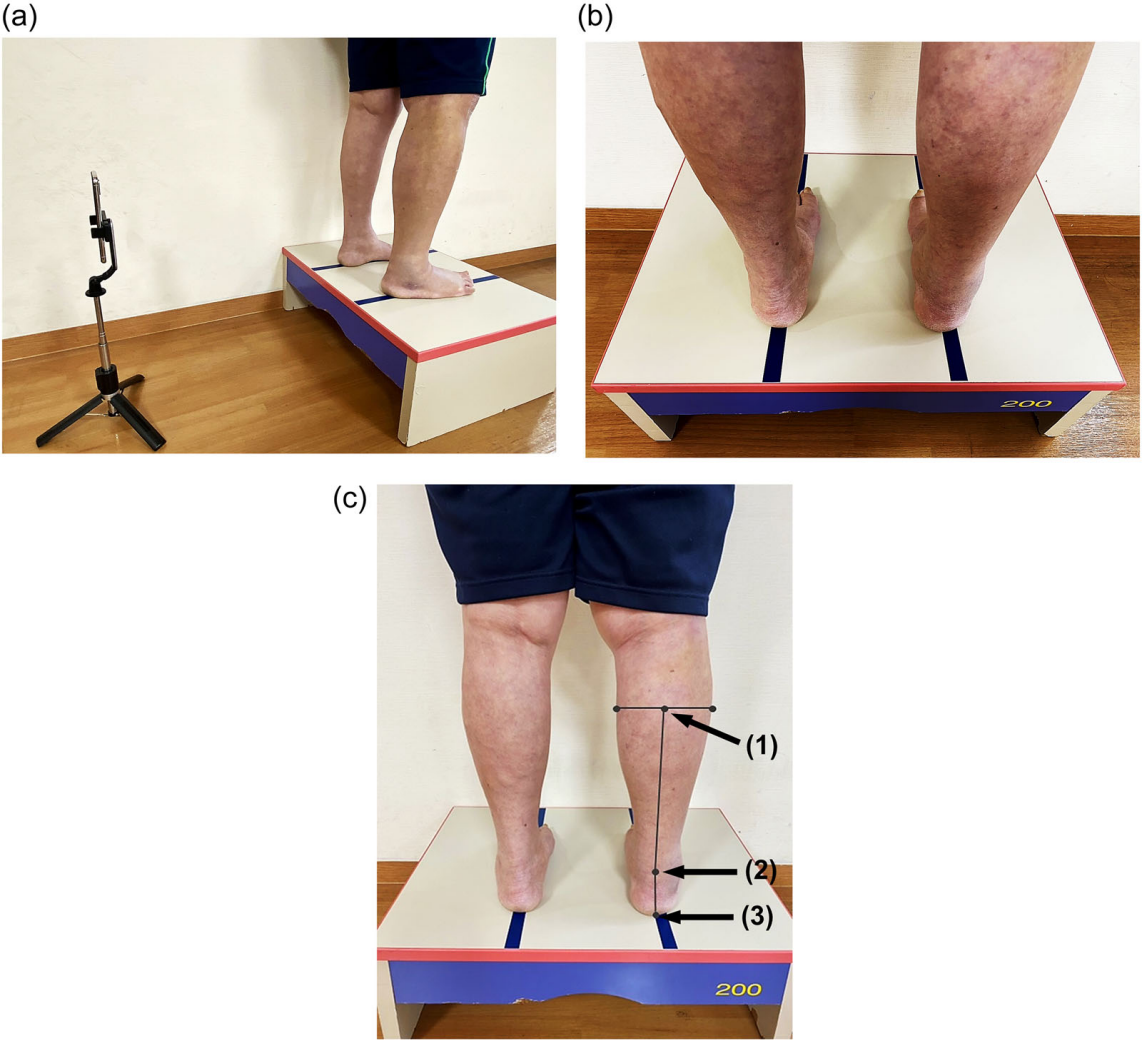
Technique for measurement of LHA. (a) The LHA was determined from behind with the patient standing on a platform 20 cm high. (b) In the standing posture, patients were instructed to align the long axis of the second metatarsal with two parallel lines marked on the platform. The distance between the two parallel lines was set at 20 cm. (c) From the photographs taken, landmarks were marked at (1) the centre of the greatest bulge of the lower leg, (2) the centre of the Achilles tendon and (3) the centre of the calcaneus bone. For the LHA definition, the angle between lines (1)–(2) and (2)–(3) was calculated using image processing software (ImageJ version 1.53). LHA was defined as minus for the varus angle and plus for the valgus angle. LHA, leg‐heel angle.

The primary outcome of this study was standing hindfoot alignment evaluated using the leg‐heel angle (LHA) [9]. The measurement technique of LHA was standardized, as shown in Figure 1. In the standing posture, patients were instructed to align the long axis of the second metatarsal with two parallel lines marked on the platform. The distance between the two parallel lines was set at 20 cm. The difference between postoperative and preoperative LHA was calculated as the amount of change in the hindfoot alignment. Secondary outcomes included (1) hindfoot complaints and (2) patient‐reported knee outcome measures determined using the knee injury and osteoarthritis outcome score (KOOS).

### Statistical analysis and sample size calculation

The amount of change in hindfoot alignment was compared between patients with severe varus knee (Group 1) and those with moderate varus, neutral or valgus knee (Group 2) using Student's *t* test and reported with the 95% confidence interval (CI) for between‐group differences. To test interobserver reliability, the intraclass correlation coefficients were calculated for two assessors. Other comparisons between the study groups were performed using Fisher's exact test for categorical variables and Student's *t* test for continuous variables. All tests were two‐sided, and *p* < 0.05 was considered significant.

In our pilot study of 20 patients, the mean ± standard deviation difference between pre‐ and postoperative LHA was 3.6 ± 4.0 in Group 1 (*n* = 8) and 2.3 ± 3.6 in Group 2 (*n* = 12). The mean ± standard deviation difference of pre‐ and postoperative LHA between groups was 2.3 ± 3.7. Based on the observed difference in the pilot study, a type I error rate of 5% and a type II error rate of 20% (80% power), a required sample size of 41 patients per treatment arm was calculated.

All statistical analyses were performed with R software (version 4.2.0).

## RESULTS

A total of 159 knees in 120 patients were included in this study. Table 1 shows patients' demographic data. Table 2 shows intra‐ and interobserver reliability for measured hindfoot alignment using the LHA.

**Table 1:** Demographic data.

	Group 1	Group 2	
	Varus correction ≥10°	Varus/valgus correction <10°	
	*n* = 52 (60 joints)	*n* = 81 (99 joints)	*p* Value
Age (years)	78.1 ± 7.2 (62–93)	75.5 ± 6.2 (54–88)	0.03*
Sex			
Female	42	60	0.41**
Male	10	21	
Body mass index (kg/m^2^)	26.3 ± 3.5 (19.9–37.1)	26.6 ± 3.7 (16.7–34.2)	0.71*
Operative side			
Unilateral	31	48	1.00**
Simultaneous bilateral	21	33	
Diagnosis			
Osteoarthritis	57	95	1.00**
Rheumatoid arthritis	3	4	
HKA angle (°)			
Preoperative	−14.3 ± 4.4 (−23.7 to −3.2)	**−**6.6 ± 5.3 (−16.0 to 10.6)	<0.01*
Postoperative	−1.0 ± 2.8 (−7.5 to 7.1)	−1.1 ± 2.9 (−8.4 to 3.8)	0.71*
Postoperative lower limb alignment (°)			
Lateral distal femoral angle	91.1 ± 1.9 (87.4–96.2)	91.1 ± 1.7 (86.9–96.7)	0.90*
Medial proximal tibial angle	90.5 ± 1.9 (86.0–96.9)	90.1 ± 1.6 (86.3–93.3)	0.21*

*Note*: Results are shown as means ± standard deviation (range).

Abbreviation: HKA, hip‐knee‐ankle.

* *p* Values were determined with Student's *t* test.

** *p* Values were determined with Fisher's exact test.

**Table 2:** Intra‐ and interobserver reliability for measured hindfoot alignment using LHA.

	LHA (preoperative)	HKA angle (postoperative)	LDFA (postoperative)	MPTA (postoperative)
Intraobserver reliability (95% CI)	0.93 (0.81–0.98)	0.98 (0.95–0.99)	0.95 (0.88–0.99)	0.94 (0.85–0.99)
Interobserver reliability (95% CI)	0.79 (0.51–0.94)	0.96 (0.82–0.99)	0.93 (0.80–0.98)	0.91 (0.65–0.98)

Abbreviations: HKA, hip‐knee‐ankle; LDFA, lateral distal femoral angle; LHA, leg‐heel angle; MPTA, medial proximal tibial angle; 95% CI, 95% confidence interval.

Table 3 shows the amounts of difference between pre‐ and postoperative LHA. The amounts were significantly larger in Group 1 than Group 2 at 2 weeks (−3.4 ± 4.0° vs. −1.7 ± 3.8°, respectively; 95% CI, −3.0 to −0.5, *p* = 0.006), 1 month (−3.6 ± 3.9° vs. −1.8 ± 3.3°, respectively; 95% CI, −3.2 to −0.3, *p* = 0.01) and 3 months postoperatively (−3.3 ± 3.5° vs. −1.7 ± 3.0°, respectively; 95% CI, −3.2 to −0.1, *p* = 0.03). No difference was observed between the two groups at 6 months postoperatively (−3.6 ± 3.1° vs. −2.8 ± 2.8°, respectively; 95% CI, −2.3 to 0.7, *p* = 0.31).

**Table 3:** Pre‐ and postoperative LHA.

	Group 1	Group 2		
	Varus correction ≥10°	Varus/valgus correction <10°	95% CI	*p* Value
LHA change from preoperative (°)				
Postoperative 2 weeks	−3.4 ± 4.0 (−12.9 to 7.6)	−1.7 ± 3.8 (−11.3 to 9.6)	−3.0 to −0.5	0.006*
Postoperative 1 month	−3.6 ± 3.9 (−12.9 to 5.5)	−1.8 ± 3.3 (−10.8 to 7.2)	−3.2 to −0.3	0.01*
Postoperative 3 months	−3.3 ± 3.5 (−11.0 to 5.4)	−1.7 ± 3.0 (−10.4 to 9.0)	−3.2 to −0.1	0.03*
Postoperative 6 months	−3.6 ± 3.1 (−10.8 to 3.2)	−2.8 ± 2.8 (−11.2 to 4.4)	−2.3 to 0.7	0.31*

*Note*: Results are shown as means ± standard deviation (range).

Abbreviations: LHA, leg‐heel angle; 95% CI, 95% confidence interval.

* *p* Values were determined with Student's *t* test.

The rates of hindfoot complaints were not significantly different between the two groups before (*p* = 0.32) or after TKA (*p* = 0.14) (Table 4). Complaints about the hindfoot before TKA were reported by two patients in Group 1; one patient reported pain around the hindfoot and one reported fatigue of the hindfoot. Eight patients in Group 2 had hindfoot complaints before TKA; four reported pain around the hindfoot, one reported fatigue of the hindfoot, one reported swelling, one reported numbness and one reported a feeling of physical disorder. Two patients in Group 1 reported numbness around the hindfoot after TKA.

**Table 4:** Number of patients with hindfoot complaints.

	Group 1	Group 2		
	Varus correction ≥10°	Varus/valgus correction <10°	95% CI	*p* Value
Hindfoot complaints				
Preoperative	2	8	0–25	0.32*
Postoperative 2 weeks	0	0	NA	NA*
Postoperative 1 month	0	0	NA	NA*
Postoperative 3 months	0	0	NA	NA*
Postoperative 6 months	2	0	0–3	0.14*

Abbreviations: NA, not available; 95% CI, 95% confidence interval.

* *p* Values were determined with Fisher's exact test.

Table 5 shows the results of pre‐ and postoperative KOOS. There were no differences between the two groups.

**Table 5:** Knee injury and osteoarthritis outcome score.

	Group 1	Group 2		
	Varus correction ≥10°	Varus/valgus correction <10°	95% CI	*p* Value
KOOS pain score				
Preoperative	46 ± 15 (14–81)	51 ± 17 (8–86)	−11 to −1	0.03*
Postoperative 2 weeks	61 ± 20 (17–92)	62 ± 18 (11–100)	−7 to 5	0.73*
Postoperative 1 month	69 ± 31 (39–100)	68 ± 32 (31–100)	−5 to 7	0.67*
Postoperative 3 months	83 ± 42 (53–100)	78 ± 39 (47–100)	−2 to 11	0.16*
Postoperative 6 months	84 ± 43 (47–100)	86 ± 44 (56–100)	−8 to 4	0.54*
KOOS symptoms score				
Preoperative	57 ± 19 (14–100)	59 ± 19 (14–100)	−8 to 4	0.54*
Postoperative 2 weeks	66 ± 21 (21–100)	66 ± 16 (25–100)	−6 to 5	0.95*
Postoperative 1 month	72 ± 31 (39–100)	70 ± 33 (14–96)	−4 to 8	0.55*
Postoperative 3 months	81 ± 42 (43–100)	78 ± 39 (32–100)	−4 to 10	0.40*
Postoperative 6 months	84 ± 43 (29–100)	84 ± 42 (57–100)	−5 to 6	0.78*
KOOS ADL score				
Preoperative	54 ± 17 (19–93)	59 ± 15 (24–99)	−10 to 0	0.06*
Postoperative 2 weeks	59 ± 19 (25–97)	61 ± 15 (18–100)	−7 to 3	0.50*
Postoperative 1 month	73 ± 32 (38–97)	74 ± 34 (19–100)	−7 to 4	0.64*
Postoperative 3 months	83 ± 42 (43–100)	81 ± 40 (41–100)	−4 to 8	0.46*
Postoperative 6 months	85 ± 43 (65–100)	86 ± 44 (60–100)	−6 to 4	0.66*
KOOS Sport/Rec score				
Preoperative	17 ± 15 (0–65)	27 ± 22 (0–90)	−16 to −3	0.004*
Postoperative 2 weeks	12 ± 16 (0–80)	16 ± 21 (0–100)	−10 to 2	0.18*
Postoperative 1 month	26 ± 24 (0–80)	30 ± 27 (0–90)	−14 to 6	0.39*
Postoperative 3 months	44 ± 31 (0–100)	46 ± 31 (0–90)	−13 to 10	0.73*
Postoperative 6 months	45 ± 30 (0–90)	56 ± 35 (5–100)	−24 to 1	0.07*
KOOS QOL score				
Preoperative	23 ± 15 (0–56)	30 ± 20 (0–88)	−13 to −1	0.02*
Postoperative 2 weeks	37 ± 26 (0–100)	41 ± 23 (0–100)	−12 to 4	0.27*
Postoperative 1 month	49 ± 29 (13–100)	46 ± 29 (0–100)	−6 to 12	0.54*
Postoperative 3 months	68 ± 38 (6–100)	61 ± 35 (6–100)	−4 to 16	0.23*
Postoperative 6 months	72 ± 39 (25–100)	67 ± 38 (0–100)	−5 to 15	0.30*

*Note*: Results are shown as means ± standard deviation (range).

Abbreviations: ADL score, functions of daily living score; KOOS, knee injury and osteoarthritis outcome score; QOL score, knee‐related quality of life score; Sport/Rec score, functions of sport and recreation score; Symptoms score, other symptoms score; 95% CI, 95% confidence interval.

* *p* Values were determined with Student's *t* test.

## DISCUSSION

The most important finding of this study was that although the amount of change in the hindfoot alignment measured by LHA was greater in Group 1 than Group 2 at 2 weeks, 1 month and 3 months after TKA, there was no significant difference between the two groups at 6 months after TKA. There were no differences between the two groups in terms of hindfoot complaints. The clinical relevance of this study lies in the potential for improved hindfoot alignment even in patients with severe lower limb deformities necessitating substantial femorotibial alignment correction. Furthermore, it is noteworthy that symptoms originating from the hindfoot are not necessarily prevalent in such patients.

While previous studies indicated that neutralizing varus femorotibial alignment with TKA can impact hindfoot alignment, the reported degree of these effects varies [1, 2, 3, 4, 5, 6, 10, 11, 12, 13]. Conflicting results have been reported regarding the correlation between the amount of change in femorotibial alignment and improvement in hindfoot alignment before and after TKA. Jeong et al. assessed hindfoot alignment with weight‐bearing radiographs and showed that the correlations between pre‐ and postoperative changes in varus alignment of the lower limb were *r* = 0.206 in terms of the heel alignment ratio defined as the ratio of the width of the calcaneus medial to the tibial axis to the total calcaneal width at its widest portion, *r* = −0.348 in terms of the heel alignment angle defined as the angle between the tibial and calcaneal axes, and *r* = −0.418 in terms of the heel alignment distance defined as the distance between the contact point of the heel and the intersection of the extended tibial axis and the distal part of the calcaneus [6]. Diao et al. measured hindfoot alignment with a long‐axial view of the hindfoot and reported that the correction of varus alignment was associated with the correction of hindfoot alignment [3]. Norton et al. investigated the change in hindfoot alignment using radiographs taken in the Saltzman hindfoot alignment view and similarly reported that the correction of varus alignment was associated with that of the hindfoot alignment [10]. Cho et al. reported that the mean postoperative change in hindfoot alignment was 4.0 ± 3.0° in patients with severe preoperative varus deformity of 10° or more, while the mean change was 1.8 ± 2.5° in patients with mild varus deformity less than 10° [2]. These results also support our findings, which showed significant differences between groups in the amount of pre‐ versus postoperative change in hindfoot alignment assessed by the LHA as a direct body measurement. On the other hand, some studies reported that although the hindfoot alignment improved after TKA, there were different relationships between preoperative severity of lower limb alignment and the degree of improvement of hindfoot alignment after TKA. Okamoto et al. reported that although postoperative knee alignment was associated with compensatory hindfoot alignment in patients with preexisting moderate knee deformities, patients with preexisting severe knee deformities experienced persistent postoperative valgus alignment of hindfoot [11].

In this study, the amounts of change in coronal plane alignment and hindfoot alignment after TKA were investigated over time. The change in LHA was larger in Group 1 than in Group 2 at 2 weeks, 1 month and 3 months postoperatively, but there was no significant difference between the two groups at 6 months postoperatively. The results of the present study were consistent with previous reports by Jeong et al. at 6 months postoperatively [6], Diao et al. at 3 months postoperatively [3] and Cho et al. at 6 weeks postoperatively [2]. On the other hand, Okamoto et al. reported a different relationship at 2 years postoperatively [11]. Therefore, the relationship between the amounts of change in coronal plane alignment and hindfoot alignment after TKA may be related to the postoperative time point.

Lee et al. reported that 35.2% (50 out of 142 patients) of preoperative TKA patients had ankle arthritis, indicating that an increase in the angle of correction of the knee joint internal rotation alignment by TKA also increases the incidence of ankle arthritis [8]. In contrast to the results of Lee et al. [8], this study showed no significant difference in hindfoot complaints between the groups. In the present study, 6.3% (10 out of 159 patients) of patients had hindfoot complaints before TKA, which was lower than the rate reported by Lee et al. [8]. This may have been due to differences in the way hindfoot complaints were determined and the occurrence of ankle arthritis was investigated.

In this study, the LHA was used to assess hindfoot alignment while patients were standing. A previous study demonstrated high reproducibility of LHA measurements in the standing position, with intra‐ and interobserver reliability scores of 0.87 and 0.90, respectively [9].

This study had some limitations. First, this was a single‐centre study. Therefore, it would be desirable to verify the results in a multicenter study to enhance external validity. Second, the sample size was insufficient to examine complications, such as ankle joint disease. Third, patients with severe valgus deformity, defined as having an HKA angle of 10° or more, were excluded from the study. Fourth, this study did not account for confounding factors that may influence postoperative hindfoot alignments, such as ankle osteoarthritis or talocalcaneal joint osteoarthritis. Fifth, although this study involved direct interviews with patients regarding hindfoot complaints, patient‐reported outcomes specifically related to the hindfoot were not measured.

## CONCLUSION

The change in hindfoot alignment was greater in Group 1 than in Group 2 up to 3 months after TKA, but no significant difference was observed at 6 months after TKA.

## AUTHOR CONTRIBUTIONS

Tsutomu Nakayama and Sachiyuki Tsukada participated in designing and performing the research; Tsutomu Nakayama, Yuya Kagami, Shingo Numajiri and Masahiro Nishino collected data; Kenji Kurosaka, Masahiro Nishino and Naoyuki Hirasawa supervised the study; Tsutomu Nakayama and Sachiyuki Tsukada wrote the manuscript and all authors checked the final version of the manuscript.

## CONFLICT OF INTEREST STATEMENT

One of the authors (ST) certifies receipt of personal payments during the study period, in an amount of less than USD 10,000 from Stryker Japan and Zimmer‐Biomet Japan.

## ETHICS STATEMENT

This study was performed following institutional review board approval. All patients provided written informed consent.

## Data Availability

The data that support the findings of this study are available from the corresponding author upon reasonable request.
